# Human Protein Tyrosine Phosphatase 1B (PTP1B): From Structure to Clinical Inhibitor Perspectives

**DOI:** 10.3390/ijms23137027

**Published:** 2022-06-24

**Authors:** Rongxing Liu, Cécile Mathieu, Jérémy Berthelet, Wenchao Zhang, Jean-Marie Dupret, Fernando Rodrigues Lima

**Affiliations:** 1 Unité de Biologie Fonctionnelle et Adaptative, CNRS, Université Paris Cité, F-75013 Paris, France; 1173942453lrx@gmail.com (R.L.); berthelet.jeremy1@gmail.com (J.B.); zhangwch611@gmail.com (W.Z.); jean-marie.dupret@u-paris.fr (J.-M.D.); 2 Faze Medicines, Cambridge, MA 02142, USA; cecilemathieu.fr@gmail.com; 3 Centre Epigénétique et Destin Cellulaire, Université Paris Cité, CNRS, F-75013 Paris, France; 4 Department of Lymphoma and Myeloma, The University of Texas MD Anderson Cancer Center, Houston, TX 77030, USA; 5 Department of Genomic Medicine, The University of Texas MD Anderson Cancer Center, Houston, TX 77030, USA

**Keywords:** PTP1B, crystal structure, inhibitors, specificity, challenge

## Abstract

Phosphorylation is an essential process in biological events and is considered critical for biological functions. In tissues, protein phosphorylation mainly occurs on tyrosine (Tyr), serine (Ser) and threonine (Thr) residues. The balance between phosphorylation and dephosphorylation is under the control of two super enzyme families, protein kinases (PKs) and protein phosphatases (PPs), respectively. Although there are many selective and effective drugs targeting phosphokinases, developing drugs targeting phosphatases is challenging. PTP1B, one of the most central protein tyrosine phosphatases (PTPs), is a key player in several human diseases and disorders, such as diabetes, obesity, and hematopoietic malignancies, through modulation of different signaling pathways. However, due to high conservation among PTPs, most PTP1B inhibitors lack specificity, raising the need to develop new strategies targeting this enzyme. In this mini-review, we summarize three classes of PTP1B inhibitors with different mechanisms: (1) targeting multiple aryl-phosphorylation sites including the catalytic site of PTP1B; (2) targeting allosteric sites of PTP1B; (3) targeting specific mRNA sequence of PTP1B. All three types of PTP1B inhibitors present good specificity over other PTPs and are promising for the development of efficient small molecules targeting this enzyme.

## 1. Introduction

PTP1B is a ubiquitous and abundant intracellular prototypic non-receptor tyrosine phosphatase that is recognized as a major negative regulator of numerous signaling cascades of metabolic and/or oncogenic relevance such as the insulin or epidermal growth factor (EGF) pathways [1,2,3]. In the case of insulin signaling, PTP1B dephosphorylates the insulin receptor (IR) as well as its substrates (insulin receptor substrates (IRS) proteins) [4]. Confirmation of PTP1B’s importance in glucose metabolism came from PTP1B knock-out (KO) mice which showed increased tyrosine phosphorylation of the IR in the liver and displayed lower blood glucose and insulin levels compared to wild-type (WT) [5]. In addition to insulin signaling, PTP1B also plays an important role in leptin signaling through dephosphorylation of JAK2, a downstream effector of the leptin receptor. This results in the inhibition of the synthesis of the appetite-stimulating hormone, neuropeptide Y [1]. In addition to its metabolic impact, several reports have pointed out the role of PTP1B in cancer serving both as a tumor suppressor or oncogene, depending on the cellular context [1,6]. In particular, mutations that affect PTP1B activity and subsequently different signaling pathways of oncogenic relevance such as the EGFR and JAK/STAT cascades were reported [1]. The fact that PTP1B can play both negative and positive roles in different signaling pathways makes it an interesting therapeutic target and has thus attracted attention for the discovery and use of PTP1B modulators in diabetes, obesity and possibly cancer [4].

As stated above, given its key functions in several signaling cascades, PTP1B activity is regulated by several mechanisms that may work in tandem to modulate the enzyme’s functions. Especially, reversible and irreversible post-translational modifications (PTMs) such as phosphorylation, oxidation, sumoylation, and proteolysis, were put forward as essential for regulating PTP1B activity [4,7,8,9,10]. Further mechanistic implications of PTP1B and its modulation can be found in the study by Yip et al. [4].

### 1.1. Structure of PTP1B

**Figure 1:**
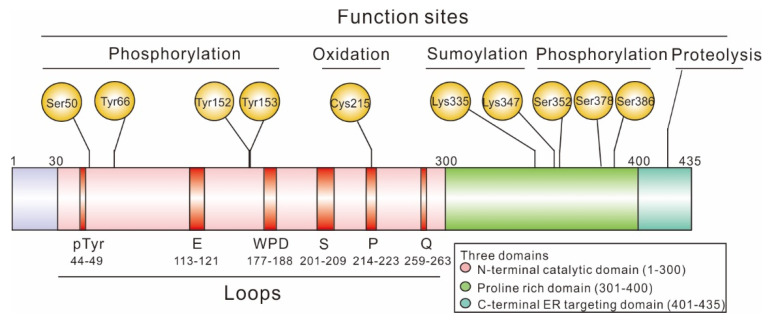
**Schematic representation of the domain structures of PTP1B full length.** Full-length PTP1B is composed of an N-terminal catalytic domain containing several important loops (1–300) and C-terminal ER targeting domain (401–435), flanking two proline-rich domains (301–400), with multiple functions at different sites of PTP1B.

**Figure 2:**
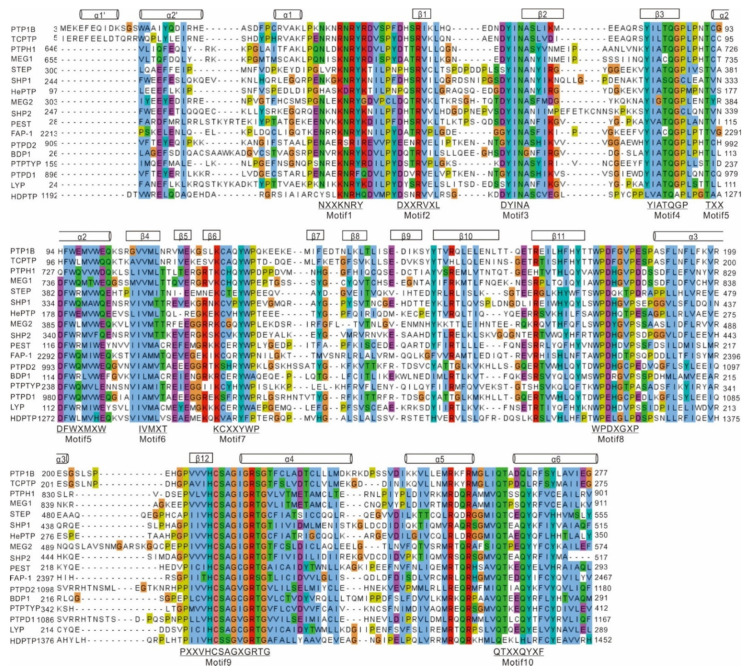
**Sequence alignment of 17 human non-receptor PTPs catalytic domains.** Sequences were obtained from Uniprot (https://www.uniprot.org/ (accessed on 7 August 2021)) database and aligned using EMBL-Clustal Omega (https://www.ebi.ac.uk/Tools/msa/clustalo (accessed on 8 August 2021)) database using default options. The alignment was further processed by Jalview (version 2.11.1.3) and colored with Clustal default option.

PTP1B is one of the major non-receptor protein tyrosine phosphatases (PTPs) whose first crystal structure was solved in 1994 [11]. At the structural level, PTP1B is composed of three domains: an N-terminal catalytic domain (1–300), a regulatory domain (301–400), and a C-terminal domain (401–435) responsible for targeting the enzyme to the endoplasmic reticulum (ER) membrane (Figure 1) [1,2]. To date, the structure of the full-length protein (1–435) has not been solved. However, truncations of human PTP1B (1–298 or 1–321) were successfully crystallized [2]. These various crystal structures all contain the first 298 amino acids corresponding to the catalytic site. This domain contains eight α-helices and 12 β-strands and shares high similarities with other non-receptor PTPs, as shown in Figure 2, where sequences from the catalytic domain of 17 non-receptor PTPs were aligned. Nevertheless, it is important to note that even though only the N-terminal catalytic domain of PTP1B was crystallized, all three domains play a critical role in the regulation of PTP1B. For more information on the structure of PTPs and notably PTP1B, we suggest the review by Tautz L. et al. [12].

#### 1.1.1. N-Terminal Catalytic Domain

The N-terminal catalytic domain of PTP1B is regulated through the PTMs of key residues found within this domain: (i) phosphorylation of crucial serine (Ser) and tyrosine (Tyr) residues, and (ii) oxidation of the catalytic cysteine (Cys) 215 in response to reactive oxygen species (ROS).

Phosphorylation within the catalytic domain of PTP1B is one of the main mechanisms resulting in the modulation of its activity. For example, phosphorylation of PTP1B at Ser50 by Akt, a Ser/Thr effector kinase involved in insulin signaling, decreases the activity of PTP1B and impairs its ability to dephosphorylate the insulin receptor in vitro [13]. In addition to Ser phosphorylation, Tyr phosphorylation of the PTP1B catalytic domain also occurs on residues 66, 152 and 153. In particular, Tyr66 was identified as one of the major sites being phosphorylated in response to insulin stimulation in vivo, leading to negative regulation of the insulin pathway [14]. Interestingly, the sequence surrounding Tyr66, _66_YINA_69_, is consistent with the consensus binding site (YXNX) for the Src homology 2 (SH2) domain of Grb2 (growth factor receptor-related protein 2) [15], which could interact with intracellular insulin receptor substrate 1 (IRS-1), thereby activating Ras and the mitogen-activated protein kinase (MAPK) pathway and regulating cell proliferation and signal transduction [16]. Moreover, mutating Tyr66 or Tyr152 and Tyr153 to phenylalanine (Phe) residues may alter the conformation of PTP1B and inhibit PTP1B binding to the insulin receptor, thereby preventing its phosphorylation by the activated IR tyrosine kinase [14].

On the other hand, oxidation of the catalytic cysteine residue of PTP1B is a key modification that was found to regulate the extent and duration of phosphotyrosine-dependent signaling response. Indeed, Cys215 oxidation abolishes PTP1B activity. Typically, due to the unique environment surrounding the active site, this catalytic Cys215 residue displays an unusually low pKa (between 4.5 and 5.5, compared to the pKa of a typical Cys, being ~8.5) [12]. Under physiological conditions (pH 7–7.5), this residue is deprotonated, resulting in increased reactivity and susceptibility to oxidation by ROS. Experiments indicated that ROS could rapidly convert the sulfenic acid (S-OH) form of PTP1B Cys215 to a cyclic sulfenamide (S-N-R), inhibiting the catalytic activity of PTP1B, but remaining a reversible modification that can be reversed by reducing agents such as reduced glutathione (GSH). However, oxidation of Cys215 to sulfinic (S-O_2_H) or sulfonic (S-O_3_H) acid inhibits PTP1B activity irreversibly [7,17].

#### 1.1.2. Regulatory Domain

The regulatory domain of PTP1B, also known as the proline-rich domain or C-terminal domain, is thought to confer substrate specificity to PTP1B. It contains two proline-rich motifs, spanning from Pro301 to Arg315, and from Ser386 to Lys397 [18], which are well-known interaction sites for Src homology 3 (SH3) domain-containing proteins [4,19], suggesting that SH3 domain-containing proteins may be a physiological target of PTP1B. Consistent with this hypothesis, the substitution of some key proline residues in the regulatory domain of PTP1B abolishes the capability of PTP1B to bind to and activate Src, although it does not impact the dephosphorylation of IR [4,19,20,21].

The regulatory domain of PTP1B harbors various PTMs, notably, phosphorylation. Many sites were identified, including Ser378, targeted by protein kinase C (PKC), and Ser352 and Ser386, that are modified by an unknown kinase in a cell cycle-dependent manner [22,23]. Several studies suggested that these sites may be modified in response to osmotic stress, and do not impair PTP1B activity [22,23]. Similarly, sumoylation was reported at Lys73, Lys335, Lys347 and Lys389 [10]. Although crystal structures of PTP1B do not include residues beyond 321, these sumoylation sites are located close to PTP1B catalytic site, suggesting that these PTMs may alter the conformation of PTP1B through steric hindrances, thereby reducing interactions with PTP1B substrates and resulting in reduced enzymatic activity [4,24].

#### 1.1.3. C-Terminal Membrane Localization Domain

In cells, PTP1B activity is associated with the ER due to the binding of the C-terminus of PTP1B to the ER membrane. It was reported that, in human platelets, cleavage of PTP1B C-terminus by calpain in vivo leads to the generation of a soluble and active enzyme, and to the concomitant changes in the pattern of protein tyrosyl phosphorylation [25]. Interestingly, disruption of calpain-1 could not only lead to an overall reduction in protein tyrosine phosphorylation in mice but also to platelet aggregation and clot retraction, emphasizing the potential of PTP1B pharmacological modulation as a therapeutic approach [4,26,27].

### 1.2. Failed PTP1B Inhibitors in Pre-Clinical or Clinical Trials

As stated previously, PTP1B serves as a master regulator of different signaling cascades of physiopathological importance, notably in obesity and type 2 diabetes [1]. PTP1B has thus attracted particular attention over the last decades as a therapeutic target and several molecules acting as inhibitors of PTP1B were studied.

**Figure 3:**
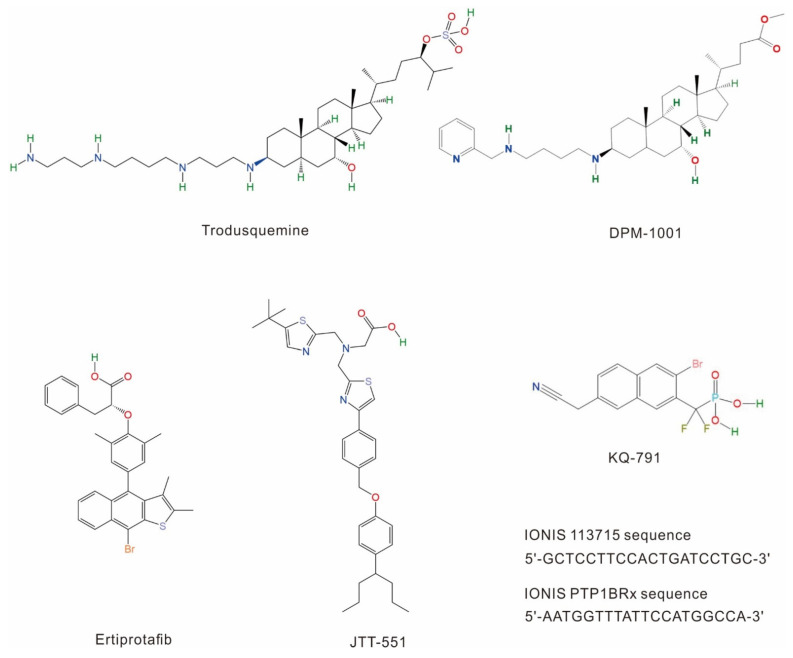
PTP1B inhibitors investigated in clinical trials.

To date, only a few PTP1B inhibitors have been tested in clinical trials (Figure 3, Table 1), including ertiprotafib (a noncompetitive multiple-action inhibitor [28]), trodusquemine (a natural allosteric inhibitor [29,30]), JTT-551 (a mixed-type inhibitor [31,32]) and other more recently identified PTP1B inhibitors [33,34]. Unfortunately, most of them were discontinued due to their insufficient efficiency, lack of specificity and notable side effects [33,35].

#### 1.2.1. Ertiprotafib

Ertiprotafib is a phosphotyrosine (pTyr) monocarboxylic acid mimetic developed by Wyeth and the first PTP1B inhibitor to be tested in clinical trials for the treatment of diabetes [36]. This inhibitor was particularly interesting due to its atypical mechanism of action. Indeed, unlike most drugs that can tightly bind to their targets and increase their melting temperature (Tm) (i.e., binding of the small molecule stabilizes the target protein), ertiprotafib lowers PTP1B Tm and destabilizes it [37,38]. To better understand the molecular basis underlying this phenomenon and develop new PTP1B inhibitors, Kumar G. S. and colleagues investigated the impact of ertiprotafib on the PTP1B structure. They found that ertiprotafib can inhibit PTP1B activity by inducing protein aggregation in a dose–response manner, providing insights into the mechanism of action of this drug. However, as we will describe later, ertiprotafib being an active site inhibitor of PTP1B, the selectivity of this compound for PTP1B (IC_50_: 1.6 to 29 µM depending on assay conditions) [28] was not as high as expected. Indeed, evidence of interaction with other proteins such as dual peroxisome proliferator-activated receptor (PPAR) alpha and gamma (EC50: 1.4 ± 0.5 µM and 1.1 ± 0.4 µM) [28] and IkappaB beta (IKK-beta) kinase (IC_50_: 400 ± 40 nM) was found [39], raising the need to develop new and more specific inhibitors.

#### 1.2.2. Trodusquemine

**Figure 4:**
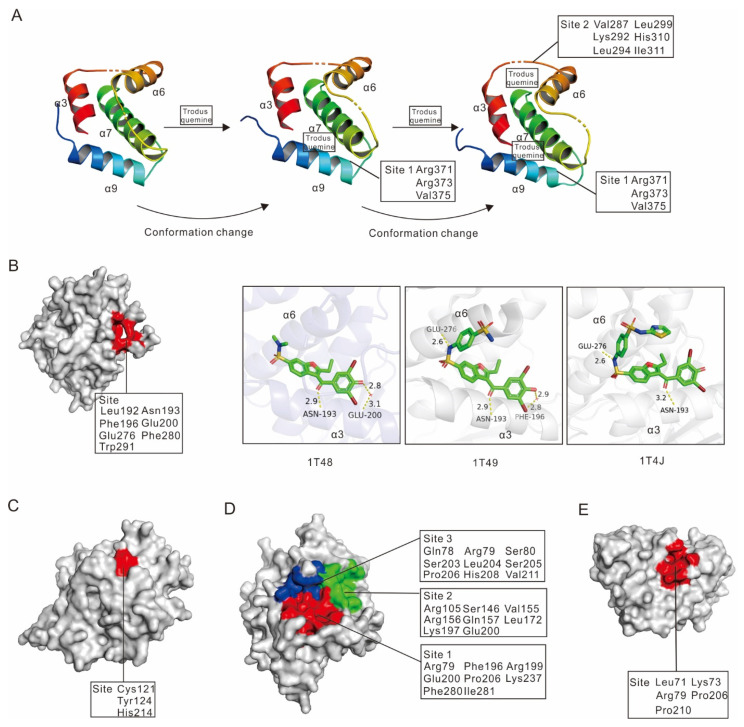
**The allosteric binding sites of PTP1B inhibitors.** (**A**) The step-by-step mechanism of PTP1B inhibition by trodusquemine. (**B**) (left panel) PTP1B was represented as surface structure and its allosteric binding site was highlighted in red; (right panel) structural representation of three inhibitors bound to the allosteric site of PTP1B (inhibitors are colored by elements; hydrogen bounds are depicted with distance in yellow dash). (**C**–**E**) PTP1B was represented as surface structure and its allosteric binding site was highlighted in color, respectively.

Trodusquemine (also known as MSI-1436), is a small molecule inhibitor that primarily binds to the PTP1B C-terminal domain in a reversible and selective manner [40]. It was first identified by Krishnan N. et al. and showed excellent specificity for PTP1B (IC_50_: 1 µM), with very little impact on T-cell protein tyrosine phosphatase (TCPTP) (IC_50_: 224 µM), the major protein tyrosine phosphatase commonly used to assess PTP1B inhibitors specificity [40]. At the structural level, trodusquemine acts as a non-competitive inhibitor of PTP1B. Two molecules of the inhibitor can bind to PTP1B in a cooperative manner and induce significant conformational changes in the protein, a mechanism that was validated by least-energy molecular dynamics simulations [32]. Indeed, trodusquemine first binds in a cavity mainly formed by Arg371, Arg373 and Val375 in α9, and then by Val287, Lys292 and Leu294 in α7 as well as residues Leu299, His310, Ile311 of PTP1B [40]. This first binding event results in a structural rearrangement of PTP1B, bringing helix α7 near the two helices α3 and α6, forming a secondary binding site [32] (Figure 4A). Therefore, this inhibitor revealed a novel allosteric binding site located within the C-terminus of PTP1B. In addition, biochemical studies showed that this small molecule largely had an increased specificity compared to previous inhibitors, reducing adverse effects. Unfortunately, due to its low activity and poor bioavailability, this inhibitor was discontinued.

#### 1.2.3. JTT-551

JTT-551, developed by the Japan Tobacco Company, is a recently developed PTP1B inhibitor. Chronic administration of JTT-551 to mice with diet-induced obesity showed anti-obesity effects, improved resistance to leptin and lipid disorders, and may also mediate glucose metabolism abnormalities [32]. Comparatively to trodusquemine, JTT-551 was reported to have good selectivity towards PTP1B over TCPTP [31,41,42]. Therefore, Fukuda S. et al. set up a series of studies to measure the enzyme inhibition constant (K_i_) of JTT-551. They found a K_i_ of 0.22 ± 0.04 μM for PTP1B, whereas values of 9.3 ± 0.4 µM were measured for TCPTP, and more than 30 µM was obtained for CD45 and LAR [31]. In addition, JTT-551 could reduce blood glucose levels and exhibit antidiabetic effects without changes in body weight in diabetic mice. All these data indicate that JTT-551 had good potential for the treatment of type 2 diabetes mellitus (T2DM) and obesity. Unfortunately, it was discontinued due to insufficient efficacy in patients and adverse effects [35].

#### 1.2.4. Other PTP1B Inhibitors Tested in Clinical Trials

Compared with Ertiprotafib, Trodusquemine and JTT-551, other PTP1B inhibitors in clinical trials were less well studied. KQ-791, developed by *Kaneq* Bioscience Company, was used to treat T2DM in Phase I clinical trials, but no reports have been released since 2019 (https://www.kanyrpharma.com/ (accessed on 20 June 2022) and https://www.clinicaltrials.gov/ct2/home (accessed on 20 June 2022)). TTP814, another PTP1B inhibitor, was tested by *TransTech* Pharma Company in Phase II clinical trials. It was discontinued in 2015 (https://adisinsight.springer.com/ (accessed on 20 June 2022)). Moreover, AbbVie/Calico Life Science declaimed that they discovered some specific inhibitors targeting TCPTP and/or PTP1B, which may be useful for the treatment of cancer or metabolic diseases, and some of them have already been in clinical trials [34].

## 2. Newly Developed Specific PTP1B Inhibitors

As we previously mentioned, it is essential to develop new selective and effective inhibitors of PTP1B to treat PTP1B-related diseases, such as T2DM, obesity, lymphoma, and breast cancer [35,43,44,45,46,47,48,49]. However, PTPs are highly conserved, making the development of such inhibitors challenging. For example, the catalytic domain of TCPTP, a protein tyrosine phosphatase essential to various cellular processes and survival in vivo [50], shares 72% sequence identity and 86% similarity with the catalytic domain with PTP1B, and thus represents a major constraint to the development of specific and effective PTP1B inhibitors [51].

In this study, we propose three strategies to specifically target PTP1B: (1) developing two-way inhibitors, binding to the catalytic site and to another secondary site that is specific for PTP1B [52,53]; (2) taking advantage of allosteric inhibitors of PTP1B; (3) using antisense oligonucleotides (ASOs) inhibitors that can selectively bind to a particular sequence of PTP1B mRNA and reduce its expression [54,55].

### 2.1. Specific PTP1B Inhibitors Targeting PTP1B A, B, C and D Sites

The catalytic site of PTP1B, also called A site, is the most accessible site within the protein. However, three additional secondary arylphosphorylation binding sites were subsequently identified and were named B, C, and D sites, respectively. Together, these four sites constitute attractive structural cavities for the creation of specific inhibitors, especially due to the presence of specific residues that are not conserved among PTPs.

#### 2.1.1. A Site

The A site is the most polar of the four sites. It is 9 Å deep (from residues Phe182 to Cys215) and 10 Å wide (from residues Tyr46 to Gln262) [52] and harbors the key highly conserved catalytic motif (motif 9: PXXVHCSAGXGRTG, Figure 2), which include the catalytic Cys215 [56,57]. Catalysis mainly relies on both the catalytic Cys215 and the flexible WPD loop (motif 8: WPDXGXP, Figure 2). This loop contributes significantly to the recognition of the peptide substrate and mediates substrate specificity [56]. Upon binding of a dianionic pTyr residue, the WPD loop interacts with the substrates and switches to a closed conformation, accompanying the movement of the substrate near the A site, then inducing catalysis of PTP1B. The catalytic mechanism of PTP1B was extensively studied at the atomic level and includes two main steps [58,59,60].

First, Cys215, which exists as a thiolate ion, acts as a nucleophile and attacks pTyr, forming a covalent thiol-phosphate intermediate [35]. The highly conserved Asp181, located in the WPD loop, acts as an acid and protonates the dephosphorylated tyrosyl group which is then released [61,62]. During this step, Arg221 forms hydrogen bonds with pTyr which maintains PTP1B in a catalytically active conformation [58]. Secondly, an activated water molecule, which establishes hydrogen bonds with the highly conserved Gln262 present in the Q-loop (motif 10: QTXXQYXF, Figure 2), reacts with the phospho-cysteine intermediate [56,63] leading to the release of inorganic phosphate and regeneration of the thiolate group of the catalytic cysteine [64].

This site, due to its primary role in PTP1B function, raised a lot of interest in the development of inhibitors. However, inhibitors that bind only to the A site result in poor selectivity especially due to the universality of the conserved A site among PTPs. Moreover, the high polarity of the A site results in many chemical constraints for the development of small molecules that often results in poor membrane permeability.

#### 2.1.2. B Site

The B site, discovered by Puius Y. A. et al., is a specific lipophilic, low-affinity, non-catalytic aryl-phosphate binding site [65]. It is adjacent to A site with 13 × 20 Å deep and almost 4 Å width, and involves mainly residues Tyr20, Arg24, Ala27, Phe52 Arg254 and Met258 - Gly259, and contributes to substrate binding and substrate specificity [52,65,66]. Indeed, among the main contacts between pTyr residue and the B site, we can note the direct interactions of the phosphate group with the guanidinium group of residues Arg24 and Arg254. These interactions allow some inhibitors to approach Phe52 and Ala27 residues, two PTP1B-specific residues, contributing greatly to their selectivity for PTP1B [53,65]. Thus, the development of small molecules establishing contacts with residues specific to the B site is a good strategy to find selective inhibitors of PTP1B [53]. Consistent with this idea, inhibitors that can interact with both the A and B sites have previously shown good specificity [67]. For example, Puius Y. A. et al. identified bis-(*para*-phosphophenyl) methane (BPPM), a synthetic non-peptide tight binding substrate with low molecular weight and high affinity (K_m_ = 16 μM for PTP1B), which can bind to both the A and B sites (PDB code: 1AAX) [65]. In the A site, BPPM establishes electrostatic interactions with Arg221, hydrogen bonds with nitrogens from the backbone chain of residues Ser216 and Arg221, van der Waals contacts with side chains of several aliphatic residues, and pi–pi stacking with residues Tyr46 and Phe182. In the B site, a second BPPM molecule interacts with residues Arg24 and Arg254, establishes weakly polar interactions with residues Met258 and Gln262, and van der Waals contacts with residues Ile219, Asp48 and Val49 [65]. More recently, Chen X. et al. designed a new compound (CD00466) that has shown excellent selectivity towards PTP1B (IC_50_ of 0.73 μM for PTP1B, IC_50_ of 22.87 μM for TCPTP) [68]. In their study, they claimed that this competitive PTP1B inhibitor could form hydrogen bonds with Arg24 and Arg254, similar to BPPM. Moreover, Met258 and Gln262 in the Q-loop also help to attract the inhibitor, thus stabilizing its binding [68].

#### 2.1.3. C Site

The C site is a charged region and is mainly formed by residues Tyr46, Arg47 and Asp48 [66]. It is wide and flat, and completely exposed to the surface of the protein, and thus to the solvent. This site is quite close to the A site and was found to be targeted by inhibitors binding to the A site. Importantly, the C site is particularly prevalent for the selectivity of inhibitors for PTP1B.

Indeed, these inhibitors take advantage of interactions such as the salt bridge and hydrogen bonds with the PTP1B-specific residue Asp48, as well as with residues Tyr46 and Arg47 [66,69]. For example, compound **3** (K_i_ = 0.29 μM for PTP1B at pH 5.5) exhibits a 40-fold increase in potency and significant selectivity for PTP1B compared to other PTPs. This is particularly due to a novel pi–pi interaction between the thiophene ring of compound 3 and Phe182, but also the basic nitrogen of the tetrahydropyridine ring that establishes a new salt bridge with the carboxyl group of Asp48 of PTP1B. These additional interactions significantly increase selectivity (PDB code: 1C88) [70]. Similarly, compound 5, another PTP1B inhibitor that associates with sites A and C (K_i_ = 39 ± 14 μM to PTP1B), displays good selectivity for PTP1B over other PTPs except for TCPTP, due to hydrogen interactions between the urea group of the compound and the carboxylic acid side chain of Asp48 (PDB code: 1NL9) [53,71]. Interestingly, to date, there are no inhibitors of PTP1B interacting only with the C site.

#### 2.1.4. D Site

The D site is a small and narrow pocket, very close to the A site and C site, and partially shielded from the solvent. It is mainly composed of polar and charged residues such as Tyr46, Glu115, Lys120, Asp181 and Ser216 [52,53]. Unexpectedly, interaction with the D site shows great superiority in the potency and selectivity of PTP1B inhibitors over other PTPs, especially over TCPTP [53]. Indeed, among all D site residues, Lys120 is identified as a determinant of selectivity between PTP1B and TCPTP [72]. Due to the significant difference between its spatial position and that of the corresponding residue (Lys122) of TCPTP, Lys120 of PTP1B is an attractive residue for the design of selective inhibitors. For example, a previous report found that adding hydroxyl or carboxylic acid groups to PTP1B inhibitors can induce more than 20-fold selectivity via interaction with this lysine [72]. Consistently, Zhang R. et al. identified a competitive inhibitor (compound 10a) by molecular-hybridization-based screening, whose dihydroxybenzene ring moiety forms hydrogen bonds with Lys120 [73]. Compound 10a achieved good selectivity towards PTP1B (IC_50_ = 0.19 μM compared to IC_50_ = 5.94 μM for TCPTP and IC_50_ = 15.86 μM for SHP2) as well as good cellular uptake in C2C12 myotubes, a common limiting factor for PTP1B inhibitors [73]. In addition, Lys120 presents numerous attractive features for the design of new drugs. Indeed, its long side chain is highly flexible and is located close to the A site. Moreover, a simple hydrogen bond donor or a carboxylic acid can form a hydrogen bond or a salt bridge with this alkaline lysine [72]. Nevertheless, the D site is narrow, small, and polar, requiring developing inhibitors with low molecular weights and reduced cell permeability [72].

#### 2.1.5. Multiple Sites Inhibitors of PTP1B

As we reviewed so far, many PTP1B inhibitors have been developed over the past decades. Most of these inhibitors target the A site and are mimics of the pTyr portion of the enzyme-substrate. However, the A site of PTP1B is both highly conserved and charged. Thus, inhibitors targeting only the A site are often neither selective nor cell-permeable. Targeting residues that are not conserved in PTPs may avoid the selectivity problems of inhibitors. Three arylphosphorylation binding sites with limited size (B, C, and D sites) are structurally close to the A site of PTP1B, meaning that most inhibitors can interact not only with the three sites alone but also with the A site. As seen previously, these three secondary sites are critical for the potency or selectivity of known PTP1B inhibitors because they differ from other PTPs by a few amino acids [74,75,76,77,78,79]. According to the extended four-site-based binding mode, PTP1B inhibitors can be mainly divided into four types (Table 2), including AC type (binding to A and C sites), AB type (binding to A and B sites), ABC type (binding to A, B, and C sites), and ADC type (binding to A, D, and C sites) [53].

Among all the residues involved in the B, C and D sites, some play critical roles in the selectivity and potency of PTP1B inhibitors. Residues Arg24 and Arg254 in the B site of PTP1B, corresponding to residues Arg26 and Tyr252 of TCPTP, are key anchors. Many moieties, such as the carboxylate moiety of salicylate, exhibit good hydrogen interaction strength with Arg24 and Arg254 residues of PTP1B, resulting in a 20-fold higher selectivity compared to TCPTP [41,72]. Similarly, in the B site, interaction with Cys32, Phe52, and Met258 residues of PTP1B, corresponding to His34, Tyr54, and Met256 in TCPTP, could largely improve the selectivity of PTP1B inhibitors [72,80]. Suitable hydrophobic moieties, such as 1,2-dithiolane, 1,3-dithian, pyrrolidine, diphenyl ether and pyridinyl group, can also form hydrogen interactions with residues Cys32, Phe52, and Met258 of PTP1B, resulting in more than 20-fold selectivity over TCPTP [72,80]. Compared to hydrogen bonding, hydrophobic interactions with the cyclic group are easier to achieve in the B site [72]. In addition to the B site, Lys120 in the D site of PTP1B, which corresponds to Lys122 in TCPTP, is also a key determinant for selectivity [72,81,82]. Lys120 is located near the A site, and only a hydrogen bond donor or acceptor group may be required to form an interaction with this lysine. Inhibitors binding to the A site could very easily interact with Lys120, also increasing the selectivity of inhibitors for PTP1B over TCPTP. Additionally, it was shown that a hydroxyl or carboxylic acid group interacting with Lys120 could help the design of more selective inhibitors [72]. Compared to the interactions with the B and D sites, the interaction network in the C site is more complex, relying mainly on a salt bridge between the inhibitor and residues of PTP1B. This is mainly because the C site is large and flat, and entirely exposed to the solvent, without a decisive factor when interacting with inhibitors. However, all these studies about specific PTP1B inhibitors relying on these four sites need to be validated by structural studies.

### 2.2. Allosteric Inhibitors

Allosteric inhibition is considered a very promising approach in the drug discovery field, especially due to a lower sequence conservation pressure than the conserved catalytic site, higher specificity, reduced adverse effects and limited toxicity [83,84]. These aspects make allosteric inhibitors very appealing when targeting proteins that share high sequence similarity and identity with others. Over the past few decades, numerous allosteric modulators of PTP1B have been reported on the ASD website (http://mdl.shsmu.edu.cn/ASD/ (accessed on 11 February 2022)). Among them, three allosteric inhibitors were co-crystallized with human PTP1B (PDB code: 1T49, 1T48, 1T4J), providing clear insights into their mechanism of action [85]. Other allosteric inhibitors of PTP1B have their mechanism of action well illustrated, but only theoretically as they have not been confirmed by structural studies.

#### 2.2.1. Allosteric Inhibitors Binding to PTP1B α3-α6-α7 Helices

The main allosteric site of PTP1B is formed by three helices: α3-α6-α7. This site is located ~20 Å away from the catalytic site and mainly involves residues Leu192, Asn193, Phe196, Glu200 on helix α3, Glu276, Phe280 on helix α6, and Trp291 on helix α7 (Figure 4B, left panel) [85].

Three noncompetitive compounds, developed by Wiesmann C. et al., target the α3-α6-α7 allosteric site of PTP1B and were co-crystallized with the protein (PDB code: 1T48, 1T49, and 1T4J, respectively) [85]. Biochemical studies showed that compound 1 is a weak PTP1B inhibitor (IC_50_ = 350 µM), whereas compound 2 (IC_50_ = 22 µM) and compound 3 (IC_50_ = 8 µM) show strong dose-dependent inhibition of this enzyme [85].

At the structural level, compound 1 only interacts with helix 3, establishing a hydrogen bond with Asn193, and interacting with Glu200 via a water molecule. Compound 2, on the other hand, interacts with both helix 3 and 6 and more particularly with Asn193, Glu276, and Phe196 (via a water molecule). Interestingly, compound 3 only establishes a hydrogen bond with Asn193 and Glu276, and no water-mediated interaction is observed (Figure 4B, right panel).

All three compounds share a benzofuran core, which could perfectly fit into the hydrophobic site formed by the three helices and block the α3-α6-α7 interactions, thus preventing the closure of the catalytic WPD loop, maintaining PTP1B in its inactive state [85]. In their molecular dynamic experiments, Li S. et al. found that compound 3 induces a conformational rearrangement of α7, disrupting the triangular interaction between α7, α3, and loop11 (residues 151–152) [86]. This leads to displacement of α3, promotion of interaction between Ser190–Tyr176, abrogation of hydrophobic interactions between Trp179–Tyr176, and impairment of the WPD loop [86]. These rearrangements ultimately modulate hydrogen bond formation to constrain the WPD loop in its open conformation [86,87,88,89,90,91], a mechanism that was confirmed in both truncated and full-length PTP1B [85].

Interestingly, some key residues involved in the allosteric binding site are not conserved in most PTPs [92], explaining for instance the important selectivity of compound 2 towards PTP1B (IC_50_ for LAR, PTP1B-ARC, and TCPTP are respectively 500 µM, 151 µM, 129 µM) [85]. This point is further confirmed in the study published by Yang Y. et al., who demonstrated that PTP1B inhibitors (H3 and H9) targeting α3–α6–α7 helices of the enzyme show evident selectivity (approximately 100 fold higher) towards PTP1B over TCPTP [93].

#### 2.2.2. Allosteric Inhibitors Binding to PTP1B α3–α6–α7–α9 Helices

As we mentioned above, trodusquemine is a natural spermine-cholesterol adduct that can take advantage of a novel allosteric binding site located within the C-terminus of PTP1B (Figure 4A). Although DepYmed discontinued its clinical trials in 2017, insights obtained from the study of trodusquemine led to the development of a new potential PTP1B inhibitor: DPM-1001 (Figure 3).

DPM-1001 shares similar inhibitory mechanisms with trodusquemine. However, several studies showed that this drug provides better results than its predecessor. Indeed, DPM-1001 is a potent inhibitor of PTP1B (IC_50_ = 100 nM) [94]. In addition, DPM-1001 is not charged, increasing its ability to cross the cell membrane and be used as a potential drug [94]. Finally, DPM-1001 binding to PTP1B differs slightly from trodusquemine. The latter interacts with the α9 of PTP1B C-terminus and then binds with α3, α6 and α7 helices, whereas DPM-1001, which is also an effective copper chelator, interacts with the C-terminus of PTP1B in an ion-dependent manner [94,95,96]. Consequently, many studies already emphasized the important efficiency of DPM-1001 as a drug in vivo and in cells. Indeed, not only does it inhibit diet-induced obesity in mice by improving insulin and leptin signaling, but also slows the growth rate of breast tumors and reduces the possibility of cancer metastasis in the lungs [94]. Nevertheless, DPM-1001 binds weakly to the catalytic domain of PTP1B, presumably by binding around the α7 helix [97].

To date, DPM-1001 is the most promising allosteric PTP1B inhibitor in terms of selectivity, efficacy, and bioavailability, and is still in preclinical trials.

#### 2.2.3. Allosteric Inhibitors Binding to PTP1B Cys121, Tyr124 and His214 Residues

Cys121 is a conserved residue, found in most PTPs and is part of the hydrophobic motif 7 (KCXXYWP, Figure 2). This site is structurally adjacent to the catalytic site, the distance between Cys121 and Cys215 being about ~8.1 Å (distance between Cα atoms) [98]. The sulfur atom of Cys121 packs against the side chain of Tyr 124, which in turn forms a hydrogen bond with His 214, thus forming a potential allosteric binding site for PTP1B inhibitors (Figure 4C) [98,99,100]. At the structural level, these three residues are critical for WPD loop mobility. Indeed, covalent modification of Cys121 was found to impair the mobility of the WPD loop, thereby preventing the formation of the active conformation of PTP1B and ultimately inhibiting the enzyme activity.

To date, most allosteric inhibitors targeting Cys121 contain a negative charge located either on α-bromoacetamide or α-fluoroacetamide group and thus form a covalent bond with Cys121, consistent with a covalent inactivation mechanism [98,99,100]. Indeed, Khan S. et al. highlighted a highly selective inhibitor, 73U, harboring an electrophilic α-bromoacetamide group that can interact with Cys121 in molecular simulations. This results in increased inhibitor-Cys121 complex stability, interruption of the overall network among Cys121, Tyr124 and His214, and protection of the highly reactive nucleophile Cys215 from covalent inhibition [100]. Similarly, Hansen S.K. et al. demonstrated that the inhibitor ABDF selectively reacts with Cys121 of PTP1B, decreasing the activity of the enzyme in vitro and in vivo, and activating the insulin signaling pathway [98]. Interestingly, Punthasee P. et al. proposed a different model, in which a noncovalent binding of an inhibitor–electrophile conjugate to the active site of PTP1B protects the highly reactive catalytic residue Cys215 from covalent modification, thus allowing inactivation of the enzyme via alkylation of the distal allosteric residue Cys121 [99].

#### 2.2.4. Allosteric Inhibitors Binding to Three Different Sites on PTP1B

Kumar A. P. et al. identified three novel allosteric binding sites and a set of small molecules that bind to these sites using fragment-based binding site mapping methods [101] (Figure 4D). The first site is surrounded by the Pro188-Glu200 helix, the Ser201-Pro210 loop, the Gly220-Arg238 helix, and the Gln78-Thr84 β-strand. The second site is surrounded by the Pro188-Glu200 helix, and the Gln78-Thr84, Leu140-Ile149, and Tyr153-Asn162 β-strands. The third site, on the other hand, is surrounded by the Gln78-Thr84 β-strand and Ser201-Pro210 loop. Common to all three sites are residues Arg79, Glu200, and Pro206, which interact with most tested ligands. In addition, the flexible Pro188-Glu200 helix, which surrounds site 1 and site 2, is essential for the allosteric regulation of PTP1B (especially residues Leu192 and Asn193), offering new opportunities for targeting PTP1B through allosteric inhibitors [59], although the structural characteristics of the three allosteric binding sites and the small molecule inhibitors binding to them have not been clearly established. In addition, it is likely that inhibitors covering multiple allosteric sites at the same time instead of a single one will have better affinity and increased specificity for PTP1B.

#### 2.2.5. Allosteric Inhibitors Binding to PTP1B Leu71, Lys73 and a Lipophilic Pocket (Arg79, Pro206 and Pro210)

In recent years, a set of 4-[(5-arylidene-4-oxothiazolidin-3-yl) methyl] benzoic acids PTP1B inhibitors were developed and showed excellent inhibition efficiency [102,103,104,105]. These effective inhibitors include two key groups: (i) a pTyr-mimetic group consisting of a thiazolidinone-coupled p-methylbenzoic acid moiety that binds to the positively charged PTP1B catalytic site through electrostatic and hydrogen bond interactions, and (ii) a lipophilic portion (formed by a differently substituted arylidene moiety) consisting of two linked aromatic rings that efficiently fits into a non-catalytic secondary phosphotyrosine binding site adjacent to the catalytic site of PTP1B. Based on this work, Ottanà R. et al. designed some new PTP1B inhibitor analogs (compounds 5 and 6) that were used in docking simulations and led to the identification of a new allosteric binding site [106].

This site is located between the β-sheet comprising residues Leu71 and Lys73 and a lipophilic pocket involving residues Arg79, Pro206 to Pro210 [106] (Figure 4E). Compound 6d, for instance, interacts within this allosteric site through hydrogen bonds with Gln78, Ser80 and Lys237, an ionic interaction with Lys237, and various hydrophobic interactions with residues from the lipophilic pocket, including Lys73, Arg79, Pro206 as well as Pro210 [106]. Interestingly, the allosteric lipophilic pocket is connected to the catalytic Cys215 by a β-strand composed of only five amino acids. It can possibly be surmised that allosteric binding could not only influence the position of this β-sheet but also sterically alter the shape of the active site [106]. Although these compounds show a good inhibitory effect on PTP1B (the maximum IC_50_ is less than 15 µM), their specificity towards other PTPs, especially TCPTP, is unknown.

### 2.3. PTP1B ASOs Inhibitors

**Figure 5:**
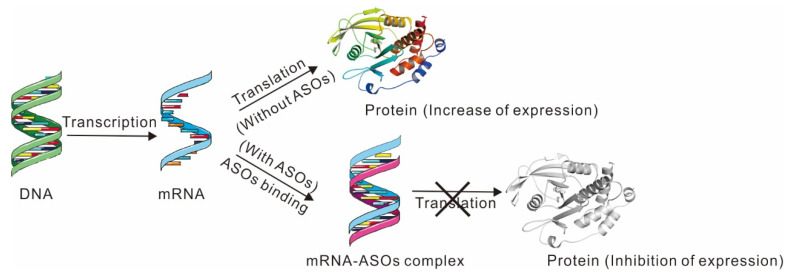
PTP1B protein expression in the presence or absence of ASOs.

ASOs generally refers to a short-stranded nucleic acid (about 15-25 nucleotides) that has undergone some chemical modifications. Its base sequence is complementary to the specific sequence of the target mRNA. After entering the cells, it can form a double-stranded structure with the target mRNA sequence, blocking protein synthesis, and finally stopping the translation machinery (Figure 5). Until now, studies on ASOs inhibitors have some limitations, such as the chemical modifications and delivery vehicles of ASOs.

To date, only two ASOs inhibitors targeting PTP1B have been designed by Ionis Pharmaceuticals, Inc. (USA): IONIS 113715 and IONIS PTP1BRx (Figure 3). IONIS 113715 is a 20-base chimeric ASOs that was identified as the most potent antisense inhibitor of PTP1B [107]. IONIS 113715 shows high binding affinity towards PTP1B mRNA sequences and induces a great improvement in insulin sensitivity, reduced expression of lipogenic genes, triglyceride accumulation in rat liver and adipose tissue, and confers nuclease resistance [107,108]. Additionally, Swarbrick M. M. et al. demonstrated that treatment with IONIS 113715 could reduce PTP1B expression in insulin-sensitive tissues in monkeys, resulting in significant improvements in insulin sensitivity, reduction in fasting insulin concentrations, and prevention of insulin resistance and T2DM associated with obesity [109]. IONIS 113715 was advanced into phase II clinical trials but was then suspended for unknown reasons and replaced by a new ASOs (IONIS PTP1BRx) currently in phase II trials. IONIS PTP1BRx presents many advantages over IONIS 113715 and may help patients who do not have a significant response to existing oral diabetes medications [110]. Indeed, IONIS PTP1BRx is a more potent inhibitor of PTP1B expression than IONIS 113715 in humans [111]. The positive effect of IONIS PTP1BRx occurs mainly through two mechanisms: (1) improvement of HbA1c levels that could improve medium-term glycemic parameters and reduce leptin signaling; (2) increase in adiponectin levels as well as significant weight reduction compared to placebo. In clinical trials, no severe hypoglycemia was observed, and no unexpected safety findings were reported [111]. However, to date, no further data have been published on this inhibitor.

## 3. Further Challenges and Perspectives of PTP1B Inhibitors

Over the past few decades, around 500 PTP1B inhibitors (including 248 phenolics, 159 terpenoids, 40 alkaloids, 24 fatty acids, and 17 steroids) have been identified/developed. They mostly interfere with the catalytic active site of PTP1B [112]. For example, PTP1B inhibitors with oxidizing properties or the ability to generate oxidative compounds, lead to PTP1B inactivation and were highly considered since oxidation of the catalytic cysteine of tyrosine phosphatases commonly leads to their inhibition [7,113]. Moreover, PTP1B is a metal-modulated enzyme, and PTP1B inhibitors containing metal ions (e.g., Zn^2+^, Fe^2+^, Cu^2+^, Cd^2+^, Mg^2+^) result in the loss of PTP1B phosphatase activity [114,115]. However, as expected, both types of PTP1B inhibitors have low specificity as they are effective against all PTPs.

Specificity is always a hurdle in drug discovery, especially for phosphatase inhibitors. The process of discovering PTP1B inhibitors has been a long journey for researchers over the past decades due to two challenges.

The first challenge is selectivity. The discovered PTP1B inhibitors act primarily on the catalytic site of the enzyme. However, the catalytic site of non-receptor PTPs is highly conserved, in particular TCPTP which shares 72% sequence identity and 86% similarity in the catalytic domain with PTP1B [51], and thus becomes the main obstacle to the development of PTP1B inhibitors. This makes it difficult for inhibitors to target a particular phosphatase subtype, leading to potential side effects and difficulties in studying pharmacological mechanisms. Two interesting strategies for the discovery of inhibitor candidates for new and improved PTP1B inhibitors were suggested. The first one relies on the use of huge chemical libraries in high throughput screening and large virtual screening [116,117]. The other strategy relies on the use of DNA encoding libraries [118] and proteolysis-targeting chimeras (PROTACs) approaches [119].

The second challenge is cellular permeability and bioavailability. The catalytic site of PTP1B is positively charged, so inhibitors acting on the catalytic site are ideally negatively charged or highly polar. However, negatively charged or highly polar inhibitors cannot effectively cross the cell membrane, and thus reach the phosphatase. Different approaches (including small molecule cargo, peptide cargo, protein cargo [120] and PROTACs [121]) to design inhibitors with improved membrane permeability could be developed based on the transportation mode. The wide distribution of the PTP1B enzyme in vivo makes it difficult to achieve efficient bioavailability using conventional drug delivery systems alone. Therefore, improved pharmacological properties and pharmaceutical dosage forms may lead the trend in discovering new PTP1B inhibitors with desirable in vivo efficacy, such as the use of nanoparticles (NPs) [122,123]. Interestingly, a recent study showed that graphene quantum dots (GQDs) carrying vanadate complex present excellent oral bioavailability, as well as high inhibitory potency and selectivity towards PTP1B [124].

Phosphorylation (by protein kinases) and dephosphorylation (by protein phosphatases) play an essential role in cellular regulation and signal transduction. Although there are many specific and effective drugs on the market targeting kinases, the development of drugs targeting phosphatases has proven to be challenging. As stated above, some inhibitors have shown an excellent inhibitory effect on PTP1B, but their study was discontinued because of their low specificity and side effects. In this mini-review, three categories of PTP1B inhibitors were reviewed with the aim of informing the inhibition mechanisms of specific PTP1B inhibitors. The cellular permeability and bioavailability of these inhibitors are still poorly understood. Therefore, other related tests, such as pharmacokinetic properties, should be further investigated.

## Figures and Tables

**Table 1:** Summary of PTP1B inhibitors (undergone/undergoing clinical trials).

Compound	Company	IC_50_ or K_i_ (µM)		Clinical Phase	Continued or Not	Indications	Trials’ Registration Number
		PTP1B	TCPTP				
Ertiprotafib	Pfizer	IC_50_ > 20	Unknown	Phase II	×	T2DM	Unknown
JTT-551	Tobacco	K_i_ = 0.22	K_i_ = 9.30	Pre-clinical	×	T2DM, obesity	Unknown
KQ-791	Kaneq Bioscience	Unknown	Unknown	Phase I	Unknown	T2DM	ClinicalTrials.gov: NCT02445911
TTP-814	TransTech Pharma	Unknown	Unknown	Phase II	×	T2DM	Unknown
Trodusquemine	DepYmed	IC_50_ = 1.0	IC_50_ = 224	Phase I	×	T2DM, obesity Metastatic breast cancer	ClinicalTrials.gov: NCT00606112 ClinicalTrials.gov: NCT02524951
DPM-1001	DepYmed	IC_50_ = 0.10	Unknown	Pre-clinical	√	T2DM, obesity	Unknown
IONIS 113715	IONIS Pharmaceuticals	IC_50_ < 0.01	Unknown	Phase II	×	T2DM	ClinicalTrials.gov: NCT00330330
IONIS PTP1BRx	IONIS Pharmaceuticals	Unknown	Unknown	Phase II	√	T2DM	ClinicalTrials.gov: NCT01918865

**Table 2:** Categories of PTP1B inhibitors on A/B/C/D sites [53].

AC type	2-(oxalyl-amino)-benzoic acid (2-OBA) derivatives
	2-carboxymethyl-benzoic acid-derived inhibitors
	difluoromethylphosphonic acid (DFMP)-based inhibitors
	isothiazolidinone (IZD)-contained inhibitors
	monocyclic thiophene-based inhibitors
AB type	monocyclic, bicyclic and tricyclic thiophene inhibitors
	sulfamic acid moiety-contained inhibitors
	other novel AB type inhibitors
ABC type	a series of OBA derivatives
	DFMP group-contained PTP1B inhibitors
	sulfamic acid moiety-contained PTP1B inhibitors
ADC type	N-(2,5-diethoxy-phenyl)-methanesul-fonamide derivatives

## Data Availability

Not applicable.

## Abbreviations

PPs, protein phosphatases; PTP1B, protein tyrosine phosphatase 1B; PTMs, post-translational modifications; TCPTP, T-cell protein tyrosine phosphatase.
